# Neuromuscular control of a five-finger pinch task is influenced by training history

**DOI:** 10.1007/s00221-025-07147-z

**Published:** 2025-09-02

**Authors:** Dylan J. Carter, James R. Forsyth, Joshua P. M. Mattock, Jonathan Shemmell

**Affiliations:** 1 Neuromotor Adaptation Laboratory, Faculty of Science, Medicine, and Health, School of Medical, Indigenous, and Health Sciences, University of Wollongong, Northfields Avenue, Wollongong, 2522 NSW Australia; 2 Biomechanics Research Laboratory, Faculty of Science, Medicine, and Health, School of Medical, Indigenous, and Health Sciences, University of Wollongong, Northfields Avenue, Wollongong, 2522 NSW Australia

**Keywords:** Synaptic input, Intermuscular coherence, Beta-band coherence, Motor units, Resistance training, Dexterity, Electromyography

## Abstract

Hand control for dexterous and strength-based tasks involves several levels of the neuromuscular system which interact to produce force. We investigated hand force, motor unit (MU) control, and intermuscular coherence in specialist-trained groups to identify how training may affect neuromuscular force control and motor neuron activity. Equal groups of ten strength-trained (rock climbers) and dexterity-trained (musicians) individuals participated. Measures of forearm muscle structure were recorded and used to normalize maximal five-finger pinch force. Several submaximal trapezoidal pinch contractions were performed while force and electromyography (EMG) were recorded, and force steadiness was calculated. Motor unit firing rates were calculated from spike trains identified by surface EMG decomposition. Intermuscular coherence was calculated between two muscles controlling the hand (the abductor pollicis brevis and flexor digitorum superificialis), providing an estimate of the common synaptic inputs to these simultaneously activated muscles. Strength-trained participants had a higher force capacity (even when normalized to muscle size) and were steadier at controlling forces. The dexterity-trained group had faster MU discharge rates in the abductor pollicis brevis muscle at moderate force levels. Measures of synaptic inputs to the motor neuron pools were inconsistent. However, the largest peaks in the beta-band were at the lowest measured force level for the dexterity-trained group, while at the highest force intensity for the strength-trained group. These findings may indicate that the neuromuscular control of hand force differs between groups with different training histories.

**Supplementary Information:**

The online version contains supplementary material available at 10.1007/s00221-025-07147-z.

## Introduction

The human hand is our most complex tool for interacting with our external environment. Hand muscles must generate a wide range of multidirectional forces to allow for both dexterous object manipulation and powerful gripping. Hand function is enabled by the mechanical characteristics of muscles in the hand and forearm, controlled by the modulation of neuronal activity within the central and peripheral nervous system. Precise activation of hand muscles is achieved by interactions between motor areas of the central nervous system, their output pathways (Lemon 2008), and the motor units (MUs) to which they project (Fuglevand 2011). When considering hand dexterity or powerful force generation, musicians and rock climbers are examples of hand function at opposite ends of the force-control spectrum. Musical training requires performers to become experts at the fine-force manipulation of their instruments while controlling muscles individually (Kimoto et al. 2019). They display increased dynamic force control and greater individuation of the fingers when compared to non-musicians (Oku and Furuya 2017; Kimoto et al. 2019). Climbers train their fingers to act synchronously, producing large muscular forces to resist their body mass against gravity (Giles et al. 2006). They have been shown to have greater maximal force capacity (Giles et al. 2006; Assmann et al. 2021) and force steadiness when compared to untrained controls (Limonta et al. 2008). Analysis of these specialized groups may reveal the aspects of the neuromuscular system which allow for these contrasting functions.

Cortical motor areas and the corticospinal tract have been strongly linked to the fine control of hand force (Sobinov and Bensmaia 2021). In non-human primates, activity in the pyramidal tract neurons of the corticospinal tract has been observed during both fine-pinch tasks and object manipulation (Quallo et al. 2012; Muir and Lemon 1983). Similarly, using functional magnetic resonance imaging, activity has been observed in cortical motor areas of humans performing dexterous tasks (Ehrsson et al. 2000), and increased excitability of the corticospinal tract has been seen following dexterity-based practice (Larsen et al. 2016). When comparing these low-force, dexterous movements to higher-force tasks, Muir and Lemon (1983) found pyramidal tract activity during a fine-force pinch was substantially reduced when the same monkey produced a powerful grip. It may, therefore, be that the inputs involved in large hand forces stem from different motor areas than those for dexterous, low-force manipulations. Activity in the pontine reticular nuclei (the origination of the reticulospinal tract) has been shown to scale linearly with increased pinch force (Danielson et al. 2024), and subcortical motor pathways have been suggested as possible mediators of early adaptations to resistance training (Glover and Baker 2020). Given that subcortical tracts typically activate muscles in coordinated, multi-joint groups (Brownstone and Chopek 2018), they may be well placed to transmit excitatory synaptic input to motor neurons when force requirements are high.

Coherence between electromyography (EMG) signals in the frequency domain, or intermuscular coherence (IMC), can be used as an indirect method of inferring the shared synaptic inputs between motor neuron pools in simultaneously active muscles (Farmer et al. 1993). Due to the low-pass filtering properties of muscle, only oscillations at low frequencies ($<10$ Hz) are involved in the production of force (Farina et al. 2014; Negro et al. 2009; Farina and Negro 2015). Coherent frequencies above this range are suggested to correlate with the high-frequency synaptic inputs to motor neuron pools, from cortical, subcortical, or spinal centers (Grosse and Brown 2003; Erimaki and Christakos 2008; Farmer et al. 2007; Boonstra et al. 2009). For example, during steady isometric and fine-force contractions, beta-band coherence (16–30 Hz) reflects shared inputs from cortical origins, with correlations between direct and indirect recordings of motor cortex activity and hand muscles observed during fine, isometric hand force contractions in monkeys and humans (Conway et al. 1995; Baker et al. 1997). Gamma-band coherence (30–60 Hz) may also be representative of cortical activity and is amplified during dynamic and high force tasks (Omlor et al. 2007; Brown et al. 1998; Brown 2000). Coherence within the alpha-band ($8-16$ Hz) has been associated with subcortical pathway activity, particularly the reticulospinal tract (Grosse and Brown 2003; Laine et al. 2021), or spinal reflex loops (Erimaki and Christakos 2008). The alpha band is the closest to the functional range for muscle contraction, and therefore these frequencies may also be transmitted to the resulting force output (Farina and Negro 2015). By comparing the coherence between active muscles during hand-force tasks in populations with dexterous or high-force training histories, we may estimate the common synaptic inputs to motor neuron pools, providing insight into the motor system.    

Further insights into the central nervous system strategies underlying force regulation can be obtained by considering MU firing activity, one of the two determinants of muscle force (along with MU recruitment) (Enoka and Duchateau 2017). Motor unit decomposition algorithms are able to discriminate individual MU action potentials from EMG recordings, allowing characterization of their spike train activity (De Luca et al. 2006; Nawab et al. 2010). The effect of resistance training on MU firing dynamics has been characterized in several studies, with increases in firing rates (Del Vecchio et al. 2019; Vila-Chã et al. 2010), or no changes observed (Sterczala et al. 2020; Casolo et al. 2021; Elgueta-Cancino et al. 2022). However, these studies have mostly investigated strength training-related changes to firing dynamics in muscles controlling the lower limb. Given that increasing force output from intrinsic hand muscles seems to be predominantly controlled by firing rates, rather than recruitment (Kukulka and Clamann 1981; De Luca et al. 1982a), the increased hand force capacity in resistance-trained individuals may be facilitated by faster discharge rates. Changes to the dynamics of firing due to dexterity-based practice are not well known. Therefore, by characterizing MU firing behavior, along with estimates of their synaptic inputs, we may improve our understanding of the central nervous system’s responses to training modalities focused on dexterous or high-force movements.

This study investigated differences in the neuromuscular system of the hand between specialized groups, trained in either fine- or high-force tasks. We hypothesized that 1) strength-trained participants (rock climbers) would produce greater maximal hand force and maintain pinch force more steadily than dexterity-trained participants (musicians) at high forces, with the inverse true at low forces (based on Limonta et al. 2008); 2) greater IMC would be observed in alpha-band frequencies in strength-trained participants, whereas greater beta- and gamma-band coherence would be observed in the dexterity-trained group; and 3) a higher MU firing rate would be observed in strength-trained participants.

## Methods

### Participants

Experimental methods were approved by the University of Wollongong Ethics Committee (#2024/073). A cohort of 20 young ($18-34$ years) healthy adults participated in the study, recruited into either a dexterity- or strength-trained group. Each group consisted of 10 participants (9 males/1 female). The recruitment criteria for the dexterity-trained participants were: (1) their main instrument must involve the individuation of digits of the dominant hand, (2) they must have performed at least eight hours of musical practice per week for the prior two years, and (3) they must have had no history of finger-specific resistance training, nor more than two hours per week of other resistance training for the previous two years. Musicians self-reported their average weekly practice time as 12.1 ($\pm 4.04$) hours/week for the prior two years. They also reported their main instrument, being guitar (5), piano (2), bass guitar (1), saxophone (1), and trumpet (1). Recruitment criteria for the strength-trained group were: 1) participants must have performed finger-related resistance training (including climbing and finger-boarding) for at least eight hours per week for the prior two years, and 2) they could have no history of regular practice of a musical instrument for the prior two years. They self-reported an average training time of 13.47 ($\pm 4.55$) hours/week of finger-based resistance training for the previous two years. They also self-reported their climbing ability (maximum boulder or lead grade) and were of an elite level according to the International Climbing Research Association scale (Draper et al. 2015). All participants were free from neuromuscular injury or impairments.

### Experimental setup

All measurements and analyses were performed on the dominant forearm of each participant, determined as the arm with which the participant would write.

#### Muscle architecture

**Fig. 1:**
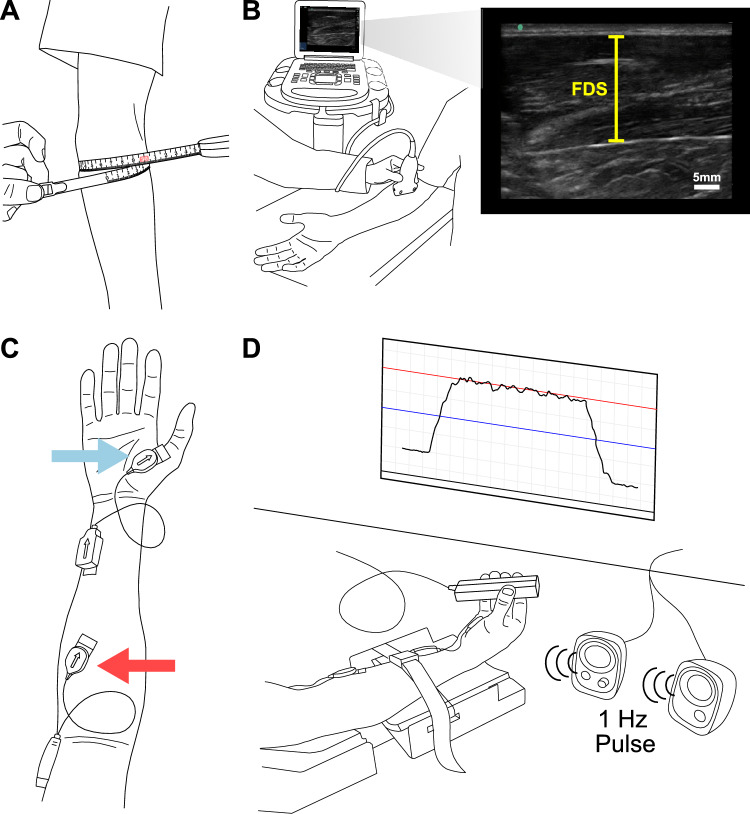
Experimental setup and tasks **A** Forearm circumference measured at the thickest portion of the forearm, following guidelines set out by the International Society for the Advancement of Kinanthropometry. **B** Ultrasonography capture of the flexor digitorum superficialis (FDS) muscle. The transducer was placed over the muscle belly of each participant’s dominant arm. An example ultrasound capture is also shown, with the muscle thickness measurement of FDS indicated in yellow, measured as the distance between the superficial and deep myofascial borders. **C** Electromyography (EMG) electrode locations. The main electrodes were placed over the abductor pollicis brevis muscle belly (indicated by the blue arrow) and FDS muscle belly (indicated by the red arrow). **D** Experimental set-up of all force tasks. The participant produced force by pinching the hand grip dynamometer, receiving real-time force feedback, with a blue guide line at 50%, and red guide line at 100% the target force for the steady period. An on-screen grid separated force generation time into 1 s segments, synchronized with a 1 Hz auditory metronome

Forearm circumference (mm) was measured using a steel tape measure (Lufkin W606PM. Michigan, USA) at the maximum girth of the forearm, using guidelines set out by the International Society for the Advancement of Kinanthropometry (Fig. 1A) (Norton 2018). Muscle thickness of the flexor digitorum superficialis (FDS) was measured via ultrasonography, using a Sonosite EdgeH2 (SonoSite, Inc., Bothell, WA, USA) with a 15–6 MHz linear array probe (maximum depth of 6 cm). Ultrasonography gel (Aquasonic Clear, Parker Laboratories. Michigan, USA) was used for acoustic coupling on the skin’s surface. Ultrasound images were collected from each participant while seated, with their forearm supported on a flat surface and fingers relaxed. Muscle location was determined as 1/3 of the linear distance between the medial epicondyle of the elbow and radial styloid process of the wrist (Abe et al. 2015), verified by palpation and visualization of live ultrasonography in a transverse view during finger flexion. The transducer was applied manually across the belly of the FDS muscle, with minimal pressure to avoid depressing the soft tissue. Two images were captured for each participant in a longitudinal view, running parallel to the muscle fibre direction (Fig. 1B). All images were analyzed using ImageJ software (National Institute for Health. Bethesda, MD, USA). Muscle thickness was measured as the perpendicular distance between superficial and deep aponeuroses (Franchi et al. 2018) (Fig. 1B) and averaged across the two images to reduce error (Koppenhaver et al. 2009). Differences between individual measurements were < 8% between images. To ensure reliability, the researcher collected and analyzed measurements of muscle thickness on a convenient sample of six participants (3 males/3 females) on two separate occasions prior to the main experiment. An intraclass correlation coefficient calculated for intrarater reliability was 0.93, indicating excellent reliability.

#### Experimental tasks

#### Electromyography (EMG)

Two wireless surface EMG sensor arrays (Trigno Galileo sensors. Delsys Inc. Massachusetts, USA) were attached to the skin over each participant’s FDS and abductor pollicis brevis (APB) muscles. These muscles were chosen as they are both active during the pinch-grip (Kerkhof et al. 2016; Blackwell et al. 1999), the target movement in each experimental task, while being sufficiently distant ($>10$ cm) to reduce crosstalk between electrodes (Hansen et al. 2005). Each array consists of two sensors: a primary sensor (with four active electrodes spaced 5 mm apart in a diamond formation) and a reference electrode (Fig. 1C). The skin was prepared following procedures outlined in the Surface Electromyography for the Non-Invasive Assessment of Muscles guidelines (Hermens et al. 1999, 2000). The first sensor was positioned over the muscle belly of the FDS, in the same position as previously identified by ultrasound (Abe at al. 2015). The second sensor was placed over the thenar eminence, at the midpoint between the radial sesamoid bone of the thumb and scaphoid tuberosity (Fig. 1C). Electromyography signals were sampled at 2222 Hz, with a bandwidth of 20–450 Hz, and transmitted wirelessly into EMGworks software (Delsys Inc. Massachusetts, USA) for storage and analysis. Baseline signal-to-noise ratio was visually monitored to ensure the collection of high-quality recordings.

#### Dexterity task

In addition to the imposed recruitment criteria, time taken to complete the Dexteria application’s “Tap it" task (Binary Labs Inc. California, USA) on an iPad (iPad Air, 5th Generation. Apple. California, USA) was recorded to provide a quantitative characterization of each group’s ability to individuate the digits. The application presents a series of targets for each finger that require an accurate screen tap response from the participant, appearing in quasi-random succession to increase difficulty. By recording the time taken to complete the series of targets, the application has been shown to identify dexterity differences in the dominant hand of healthy individuals across age groups (Kizony et al. 2016). An initial calibration step was undertaken for finger position, accounting for anatomical variation. The first two levels of the application were used to familiarize participants, with each level repeated twice. On the application’s third level, the time taken to complete the task, accuracy and number of trials to reach a score of >95% accuracy were recorded.

#### Five-finger pinch task

Five-finger pinch force was measured using a pinch-grip force transducer (ST Grip Force Transducer, model MLT004. ADInstruments, Dunedin, New Zealand), collected into a PowerLab Data Acquisition System (Model 26T. AD Instruments, Dunedin, New Zealand). Participants were asked to grip the device between the distal pads of the first phalange and phalanges two to four, with a small amount of wrist flexion (Fig. 1D). No physical restraints of the hand or wrist were employed, and thus small movements of these joints were possible. This task required the contraction of all digits, which given the compartmentalization of the FDS (Fleckenstein et al. 1992), ensured that the EMG electrodes captured muscle activity data from an active FDS compartment of each participant regardless of anatomical variation. The task was chosen to be somewhat familiar to participants from both groups, reminiscent of the forceful pinching involved in rock-climbing, or gripping an instrument with all fingers (during instrument manipulation or stabilization). Force traces were displayed in real-time on LabChart software (Version 8. AD Instruments, Dunedin, New Zealand). Force and EMG data were time-synchronized via a common voltage trigger.

A standardized force warm-up was implemented before force testing, consisting of several sub-maximal efforts. Participants were seated with their arm restrained in a custom-made forearm stabilization unit (Fig. 1D) for all force tasks. Maximal voluntary contraction (MVC) was measured by three maximal pinch contractions consisting of 3 s force efforts, with 2 mins rest between attempts. Researchers gave loud verbal encouragement during each contraction. The maximal force produced during the maximal efforts was recorded as the participant’s MVC (N).

Participants were then asked to perform several submaximal, trapezoidal contractions while EMG was recorded. Force targets were normalized for each participant as a percentage of their MVC, at 15, 35, 55 and 70% (similar to Casolo et al. 2021). Two guide lines were programmed in LabChart software at 50% and 100% of the target force (Fig. 1D). Participants were asked to produce each force contraction in the following method: a 2 s ramp to the target force, followed by a 10 s steady isometric contraction (keeping the force trace as close to the target as possible), followed by an inverse ramp to rest over 2 s. To assist with the timing of the protocol, a 1 Hz, time-synchronized metronome beat was produced, along with an on-screen grid with 1 Hz vertical gridlines. Each force target was repeated three times, with 2 min rests between each contraction, for a total of twelve trials per participant. Force levels were completed in order from lowest (15% MVC) to highest (70% MVC) intensity.

### Data analysis

Data analyses were undertaken using MATLAB software (Version R2024A. The MathWorks Inc. Natick, Massachusetts). Data were analyzed both with and without the inclusion of female data, due to the low number of female participants recruited to each group. Given that the omission of female data did not substantially alter interpretations, the full data set is presented. Force steadiness was quantified as the coefficient of variation (COV) in relation to the target force for each submaximal trial (Blomkvist et al. 2018), measured over the center six seconds of the steady period (Fig. 1D).

Electromyography data captured during the submaximal force tasks were decomposed into individual MU action potentials using NeuroMap software (Delsys Inc. Massachusetts, USA) (De Luca et al. 2015, 2006). Accuracy scores of spike trains are computed within the software (Nawab et al. 2010), and have been considered comparable to those obtained using more comprehensive methods, such as spike-triggered averaging, when they exceed 90% (Herda et al. 2020). Any spike trains with an accuracy below this threshold were, therefore, excluded. The remaining spike trains were processed using custom-made MATLAB scripts. Motor unit firing rates were calculated from each spike train during the 4 s of force data that contained the smallest COV, to avoid firing rates skewed by sudden adjustments in force. This was determined by a sliding window of 4 s bins moved at 50 ms increments over the force data of each submaximal trial. The resulting spike trains were filtered, with any identified MU trains containing less than seven interpulse intervals (IPIs) removed, along with those considered to be irregularly firing (COV of IPIs$(COV_{IPIs})>30\%$; Holobar et al. 2010). Average firing rates were calculated as $\bar{x}(\frac{1}{IPIs})$ during this 4 s period. Firing thresholds were calculated as the force corresponding to the onset of regular firing for each MU, normalized as a percentage of the participant’s MVC. The first IPI followed by a 750 ms period of $COV_{IPIs}<30\%$ was considered the start of regular firing (a method similar to Jesunathadas et al. 2010).

Intermuscular coherence was calculated from the first channel of FDS and APB EMG collected during submaximal contractions. The same 4 s period used for calculating average MU firing rate was used for IMC estimation. This allowed for comparison between the two measures, while reducing the possibility of visuomotor afferent information caused by changes in the on-screen force trace being represented within coherence estimates (Laine et al. 2014). Each 4 s window of EMG data was high-pass Butterworth filtered at 20 Hz. High-pass filtering allows for less overlap between the MU action potentials, thereby increasing the low-frequency resolution (Laine and Valero-Cuevas 2017; Boonstra and Breakspear 2012), while removing noise and movement artifacts. The data were normalized to unit variance (*z*-scores), allowing for pooling of coherence estimates between participants (Halliday and Rosenberg 2000). The EMG signal was subsequently rectified and enveloped by computing the absolute value of the signal’s Hilbert transform. (Boonstra and Breakspear 2012).

Coherence was calculated using the “sp2a2.m1” Type 0 routine from the *NeuroSpec* MATLAB toolbox (Amjad et al. 1997; Halliday et al. 1995; Rosenberg et al. 1989; Halliday and Rosenberg 2000). Electromyography signals were decomposed into their auto- and cross-spectrum using the Discrete-Fourier Transform and a sliding window of non-overlapping 460 ms bins. Coherence between each signal was calculated, with the coherence function ($R_{xy}$) at frequency $\lambda$ being defined as:

1 $$\begin{aligned} |R_{xy} (\lambda )|^2 = \frac{|f_{xy} (\lambda )|^2}{f_{xx} (\lambda ) f_{yy} (\lambda )} \end{aligned}$$

**Fig. 2:**
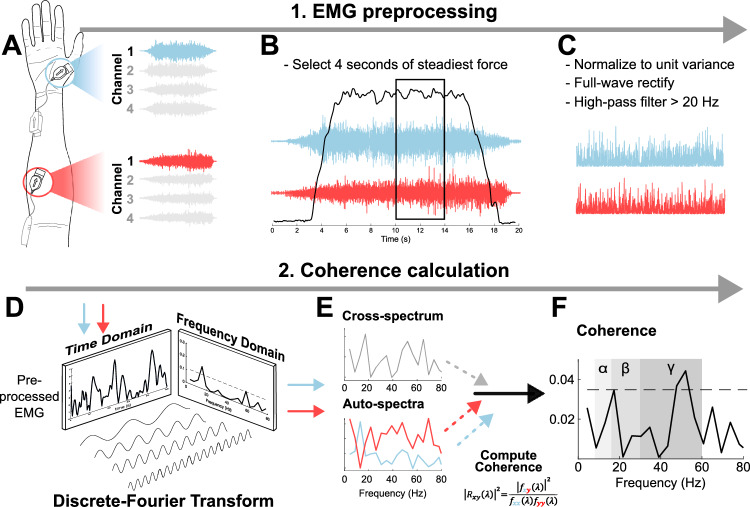
Intermuscular coherence analysis. **A** Four channels of electromyography (EMG) were captured per muscle, with channel one used for coherence analysis. **B** The steadiest 4 s period of force, determined as the period with the lowest coefficient of variation in relation to the target force, was selected using a sliding window of 50 ms increments. **C** The clipped 4 s of EMG signal was high-pass filtered (> 20 Hz), normalized to unit variance and full-wave rectified. **D** A visual representation of the Discrete Fourier Transform, converting each 460 ms bin of data from the time to frequency domain, resulting in the cross-spectrum and auto-spectra. **E** The identified spectra were compared in the frequency domain to calculate coherence ($R_{xy}$), using the displayed formula. Lambda ($\lambda$) represents a given frequency, $f_{xx}$ and $f_{yy}$ represent the auto-spectrum of each signal, and $f_{xy}$ the cross-spectrum. **F** Coherence estimates are presented at each identified frequency. The coherence bands are represented by gray shading, with alpha- ($\alpha$), beta- ($\beta$) and gamma-bands ($\gamma$)

Whereby, $f_{xx} (\lambda )$ and $f_{yy} (\lambda )$ represent the auto-spectrum of inputs *x* and *y*, with the cross-spectrum defined as $f_{xy} (\lambda )$ (Halliday et al. 1995). Coherence provides a measure of correlation between signals in the frequency domain, with values ranging between 0 (meaning the signals are completely discrete) and 1 (perfectly correlated). Coherence scores were pooled across participants in each group using the method from Amjad et al. (1997) at each discrete force level. Coherence data did not contain high estimates ($>0.8$) of IMC across all frequency bands, which would be representative of data with substantial cross-talk between electrodes (Nojima et al. 2018; Hansen et al. 2005) (Fig. 2).

An additional, post-hoc analysis was undertaken to investigate visually observed differences in FDS EMG activity between participants. To identify these differences in muscle activity, the smoothed power of the EMG signal for each muscle was calculated across participants at each force level. The first channel of recorded EMG from each muscle was *z*-normalized, full-wave rectified, and smoothed using a 500 ms sliding window calculating the root-mean-square. The first 15 s of each submaximal trial was analyzed, as this period contained the initial ramp period of the trapezoidal contraction, where differences were visually observed during data analysis. This period also excluded large changes in EMG amplitude during the ramp-down period, which may obscure results. The smoothed power of the EMG signal was subsequently averaged across trials for each participant at each force level, yielding a representation of the average muscle activation pattern for each participant during each submaximal contraction intensity.

### Statistical analysis

Statistical analyses were undertaken using R (version 2024.04.2+764; Vienna, Austria), and MATLAB software. A bootstrapping approach was implemented for hypothesis testing, using the *boot* (Canty and Ripley 2024; Davison and Hinkley 1997) and *bootES* (Kirby and Gerlanc 2013) packages in R. A total of 10,000 bootstrapped samples (with replacement) were generated for each comparison. This method provides a robust method of statistical inference, especially when sample sizes are small and parametric assumptions underlying *t*-tests may not be satisfied (Carpenter and Bithell 2000). The bootstrapped samples were used to build 95% confidence intervals (CIs) for group mean differences and effect sizes. Due to the nature of this statistical approach, no *p*-values are reported. Instead, the results of each 95% CI are interpreted as significant when an absence of overlap between the upper and lower bounds is observed. All effect sizes are presented as Cohen’s D, classified as trivial when $d < 0.2$, small when $d = 0.2 - 0.5$, moderate when $d = 0.5-0.8$, and large when $d \ge 0.8$ (Cohen 1988). Linear regression analyses were conducted to examine the relationship between maximum force and the COV of force at each contraction intensity, using the *stats* package in R (R Core Team 2024). For each regression model, the adjusted R$^2$ value, intercept, and slope coefficient with its corresponding 95% CI were calculated. Model assumptions were visually assessed using the *performance* package in R (Lüdecke et al. 2021)

Motor unit average firing rates were analyzed using a linear mixed effects model, implemented via the *lme4* package in R (Bates et al. 2015). Given the number of identified MUs differed for each participant, mean-comparison statistical tests are inappropriate. Furthermore, we cannot assume the homogeneity of identified MUs across participants, as the most readily identified MUs are both superficial and large (Farina and Holobar 2016). Participant means would, therefore, represent a sample of heterogeneous MUs with large variances in firing rate. By incorporating a mixed-effects structure into the analysis, it is possible to account for some of the known sources of variation in the data. The formula for the linear mixed-effects model was:

2 $$\begin{aligned} & \text {MU}_{\text {Firing Rate}} \sim \text {Testing Group} * {\%}_\text {MVC} * \text {Muscle} \nonumber \\ & \quad + (1|\text {Participant}) + (1|\text {Firing Threshold)} \end{aligned}$$

A random intercept was included for each participant to account for inter-participant variability (which may include skin thickness, subcutaneous fat tissue and electrode placement). A second random intercept was included for firing threshold, as each MU’s firing rate is correlated with its firing threshold (Henneman’s “Size Principle” (Henneman 1985)). Smaller MUs, which are recruited at lower forces, fire faster than larger, higher-threshold MUs (De Luca et al. 1982b; De Luca and Contessa 2012; De Luca and Hostage 2010). By accounting for random variability caused by differences in MU size, a more representative measure of group differences in firing rate can be observed (Sterczala et al. 2020). Model assumptions and fit were visually assessed using the *performance* package in R (Lüdecke et al. 2021). To evaluate differences between groups for each force level and muscle separately without interactions, *a priori* pairwise comparisons were conducted using the *EMmeans* package in R (Lenth 2024). For consistency, the estimated marginal means (EMM; $\hat{Y}$) and 95% CIs are reported.

Pooled IMC estimates from the EMG signals for each group are represented graphically, along with the percentage of trials with significant coherence at each identified frequency. The pooled coherence score for an individual frequency is considered significant when the estimate is greater than the 95% confidence limit (CL). The CLs were calculated from the number of segments (*L*) included in each coherence estimate, using the method from Halliday et al. (1995):

3 $$\begin{aligned} CL = 1 - (0.05)^{1/L-1} \end{aligned}$$

To assess whether pooled coherence estimates contained trials exhibiting significant differences in coherence, a $\chi ^2$ difference of coherence test was implemented. Between-group comparisons of pooled IMC were also analyzed, with upper and lower 95% CIs calculated using the $\chi ^2$ difference of coherence test in Amjad et al. (1997). All coherence analyses were implemented using the *NeuroSpec* toolbox.

Post-hoc analysis of muscle activity during the ramp phase of each submaximal five-finger pinch contraction was implemented in R. Clusters of participants with similar smoothed EMG activity were identified using the *dtwclust* package (Sardá-Espinosa 2019) and its “k-shape clustering" algorithm. The appropriate number of clusters (*k*) were identified using a two-step method. First, the Within-Cluster Sum of Squares (WCSS) method was implemented to produce an elbow plot, which was visually inspected to determine whether clustering was appropriate. Secondly, if clustering was identified as appropriate, silhouette scores (SIL) for ranges of *k* between 2 and 5 were calculated, with the largest value considered the optimal number of clusters. Representative results of the clustering analysis are presented.

All materials used for data and statistical analyses are available at the GitHub repository: https://github.com/dyljcarter/repo-carter-et-al-2025.

## Results

### Functional anatomy

Anthropometric and demographic data are presented in Table 1.

**Table 1:** Participant anthropometric and demographic data

	Dexterity-trained		Strength-trained	
Measure	Mean	±SD	Mean	±SD
Age (years)	23.00	5.27	23.70	4.06
Height (cm)	182.80	7.26	174.04	5.94
Mass (kg)	74.99	9.22	67.41	8.66
Hand training time (hrs/wk)	12.10	4.04	13.47	4.55

Values shown as means (± standard deviation (SD))

The groups differed in forearm circumference, with the strength-trained group ($\bar{x} = 276.50$ mm) having on average a 12.30 mm (95% CI [$-3.54, 25.79$]) larger forearm circumference than the dexterity-trained group ($\bar{x} = 264.20$ mm). This gave a moderate effect size of $d = 0.72$ (95% CI [$-0.44, 2.08$]). In contrast, FDS thickness was an average of 3.46 mm (95% CI [$-21.95, 29.95$]) greater in the strength-trained group ($\bar{x} = 195.11$ mm) than dexterity-trained ($\bar{x} = 191.65$ mm). The size of this difference was trivial ($d = 0.11$, 95% CI [$-0.86, 1.07$]).

### Dexterity

There were large ($d = 1.01$, 95% CI [0.08, 1.82]) between-group differences in time to complete the dexterity task, with the dexterity-trained group ($\bar{x} = 15.47$ s) being on average 2.07 s (95% CI [0.39, 3.90]) faster than the strength-trained group ($\bar{x} = 17.54$ s). There were little to no differences in task accuracy, which we constrained, with the strength-trained group being $<1$% more accurate (95% CI [0.38, 2.17]). There were also no differences in the number of attempts taken to reach 95% accuracy (median difference = 0).

### Force control

**Fig. 3:**
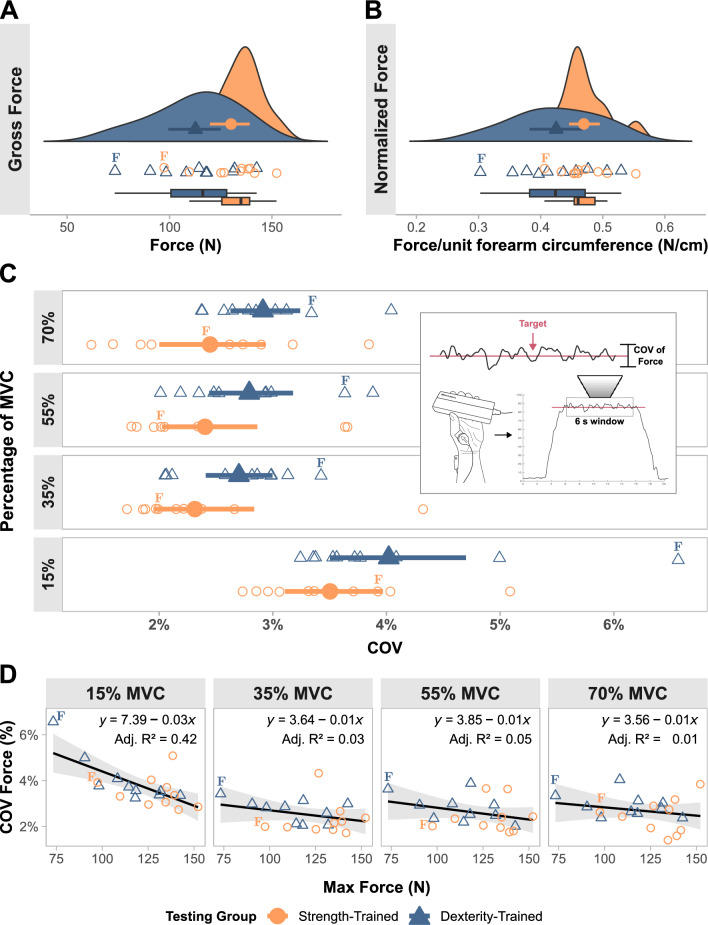
Differences in force capacity and control. **A** Between-group differences in gross force capacity measured using a maximal pinch force task. **B** Gross force capacity normalized to each participant’s forearm circumference, providing an estimate of force per unit muscle. **A and B** Group means and bootstrapped 95% confidence intervals (CI) are shown within their respective kernel density plots. Directly below, individual participant data is shown. Box and whisker plots show medians and interquartile ranges. **C** Bootstrapped means and 95% CIs of the coefficient of variation (COV) of force, measured during the center 6 s steady portion of trapezoidal contractions. Inset schematic shows the method of calculating the COV. Data from each force target is presented in separate subplots. **D** Linear regression analyses modeling the relationship between gross force and COV of force. Each force level is presented as separate subplots, with adjusted R$^2$ values (Adj. R$^2$) and linear regression equations. **A–D** Data collected from female participants are tagged as “F"

The strength-trained group produced an average peak force of 130.02 N (Fig 3A), 17.38 N (95% CI [1.57, 33.41]) greater than the dexterity-trained group ($\bar{x} = 112.64$ N) in the maximal force task, showing a large effect ($d = 0.93$, 95% CI [$-0.04, 1.89$]). When peak force was normalized to forearm circumference, the difference in group means was moderate ($d = 0.77$, 95% CI [$-0.22, 1.70$]) (Fig 3B). Peak force also differed moderately between dexterity and strength-trained groups when normalized to FDS thickness ($d = 0.83$, 95% CI [$-0.14, 1.65$]). All force COV measurements were calculated in relation to the target force, rather than the mean. The dexterity-trained group had slightly greater COV across all force levels, with moderate effect sizes (15% MVC: $d = 0.58$, 95% CI [$-0.44, 1.30$]; 35% MVC: $d = 0.61$, 95% CI [$-0.51, 2.09$]; 55% MVC: $d = 0.60$, 95% CI [$-0.41, 1.69$]; 70% MVC $d = 0.72$, 95% CI [$-0.29, 1.65$]). The mean percentage of COV and bootstrapped 95% CI are presented in Fig. 3C. Assumptions for the linear regression analyses were visually assessed and found to be reasonably satisfied. The results of each linear regression analysis, modeling the relationship between peak force (N) and COV of force (%) at each force level, are presented in Fig. 3D, along with the corresponding adjusted R$^2$ values and regression equations. The slope coefficients were, at 15% MVC: $b = -0.03$ (95% CI [$-0.05$, $-0.01$]); at 35% MVC: $b = -0.01$ (95% CI [$-0.02$, 0.01]); at 55% MVC: $b = -0.01$ (95% CI [$-0.03$, 0.01]); and at 70% MVC: $b = -0.01$ (95% CI [$-0.02$, 0.01]).

### Motor unit average firing rate

**Fig. 4:**
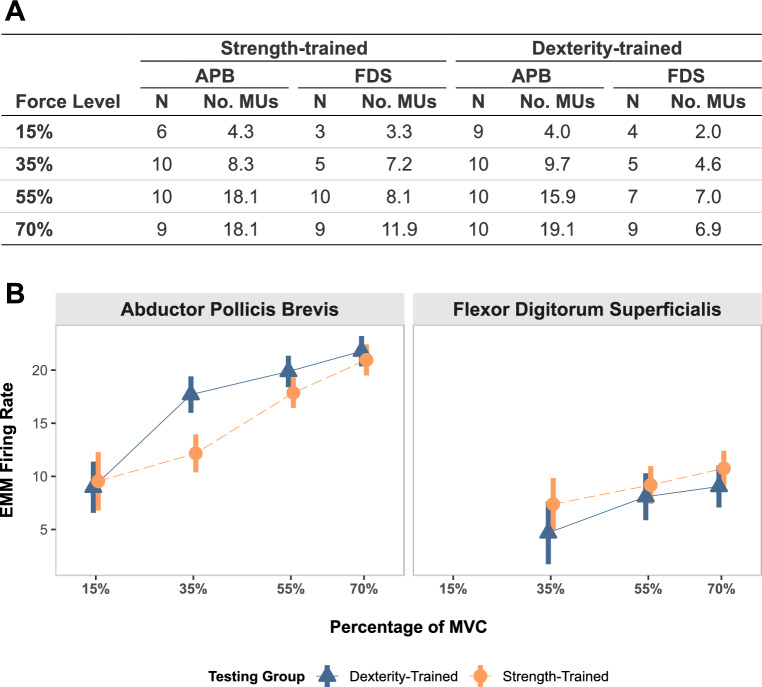
Number of motor units identified and their average firing rates for the abductor pollicis brevis (APB) and flexor digitorum superficialis (FDS) muscles. **A** Table of the average number of identified motor units for each muscle (APB and FDS) and force level, separated by testing group. For each force level, the number of participants with identified motor units is reported (N), along with the average number of motor units identified per participant across the three submaximal trials (No. MUs). **B** Estimated marginal mean (EMM) motor unit firing rates are presented for each force level, with error bars representing their 95% confidence intervals (CI). Data from each muscle (APB and FDS) is presented in a separate subplot. Data from 15% MVC in the FDS is not displayed, due to the low number of identified motor units

A total of 1312 identified MUs met our inclusion criteria. The number of participants with identified MUs at each force level, along with the average number of identified MUs per participant are presented in Fig. 4A. In the comparison of MU firing rates, assumptions for the linear mixed-effects model were met, including normality of residuals and homoscedasticity. The model exhibited a strong overall fit, with a Conditional $R^2$ of 0.67, indicating that both fixed and random effects explain 67% of the variance in the data. The Marginal $R^2$ was 0.39, reflecting the proportion of variance explained by the fixed effects alone. Due to the low number of identified MUs for the FDS at the 15% MVC level, group comparisons of this trial are not reported. However, the data from this trial were still included in the model to increase its robustness. The results from FDS at 35% MVC and APB at 15% MVC are reported. However, they also contained a small sample of data points from only a subset of participants in each group, and thus their comparisons should be interpreted cautiously.

In the APB muscle at 15% MVC, there were no observable differences in the EMM firing rate between groups (dexterity-trained: 8.98 Hz; strength-trained: 9.54 Hz). At 35% MVC in the APB, the dexterity-trained group’s EMM firing rate (17.69 Hz) was estimated to be 5.53 Hz (95% CI [3.06, 8.00]) faster than the strength-trained group (12.17 Hz). A smaller, but similar difference in APB firing rate was seen between dexterity-trained (EMM $= 19.87$ Hz) and strength-trained (EMM $= 17.86$ Hz) participants at 55% MVC ($\hat{Y} = 2.01$ Hz, 95% CI [$-0.03, 4.05$]). At 70% MVC in the APB, no between-group differences were found (dexterity-trained: EMM $= 21.78$ Hz; strength-trained: EMM $= 20.96$ Hz) (Fig. 4B).

Despite marginal increases in firing rates at each force level in the strength-trained group, no differences were detected between groups in the FDS muscle. Estimated marginal mean firing rates for the FDS in the dexterity-trained groups were, at 15% MVC: 5.46 Hz; at 35%: 4.68 Hz; at 55%: 8.08 Hz; and at 70%: 9.04 Hz. In the strength-trained group, FDS EMM firing rates were, at 15% MVC: 5.40 Hz; at 35%: 7.38 Hz; at 55%: 9.18 Hz; and at 70%: 10.75 Hz.

### Coherence

**Fig. 5:**
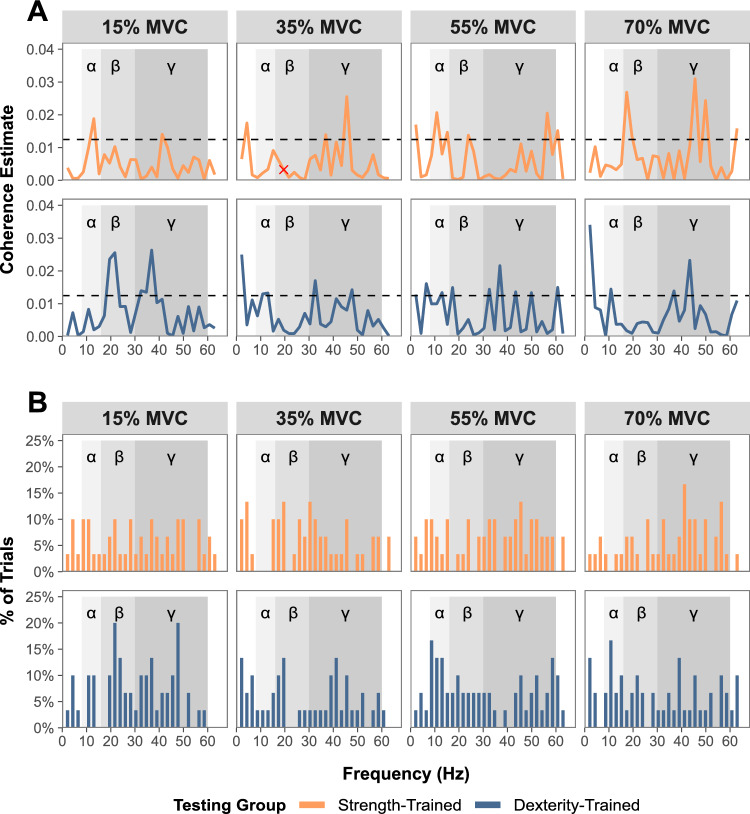
Pooled intermuscular coherence (IMC). A composite figure of IMC estimates at each force level, being 15, 35, 55, and 70% of maximal voluntary contraction. **A** Pooled coherence estimates for each group are presented. Frequencies crossing the confidence limit (horizontal dashed line) are representative of significant IMC. Any frequencies with significant within-group differences of coherence when pooled, identified using the $\chi ^2$ difference of coherence test (Amjad et al. 1997), are marked with an “X". **B** The percentage of individual trials with significant coherence at each identified frequency (frequency resolution of 2.17 Hz) are presented for each group. **A** and **B** Coherence bands are represented by gray shading, with the lightest shade identifying alpha ($\alpha$), and successively darker shades indicating beta ($\beta$) and gamma ($\gamma$) frequency bands

A total of 96 Discrete Fourier Transform segments (8 segments per trial), with a frequency resolution of 2.17 Hz were identified per participant. The results of pooled IMC estimates are presented in Fig. 5. The list of individual frequencies with significant coherence for each testing group are presented in Online Resource 1.

**Fig. 6:**
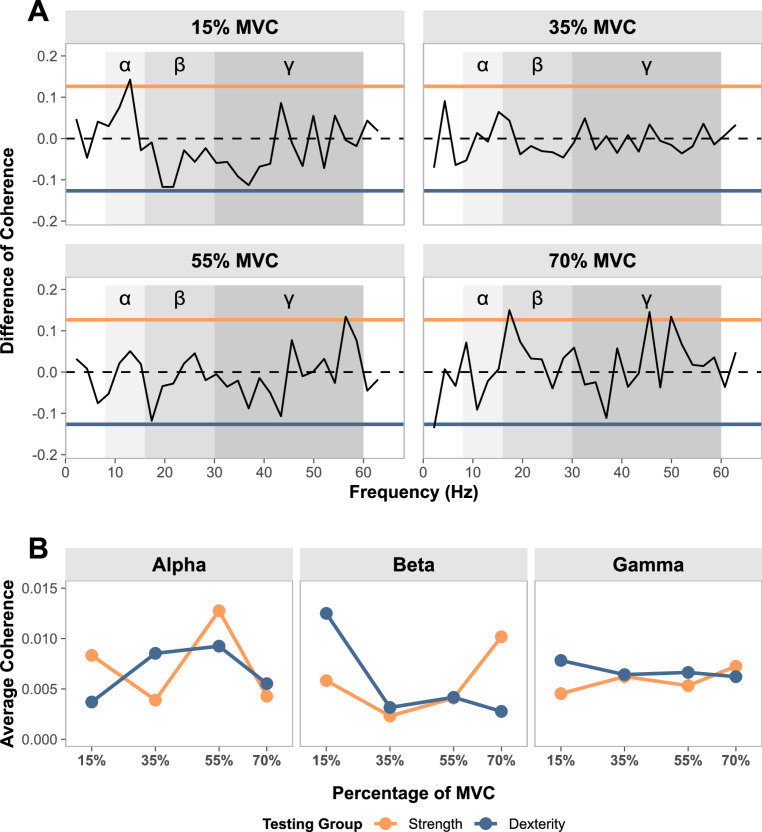
Between-group differences in intermuscular coherence (IMC). **A** Comparison of coherence test (Amjad et al. 1997), displaying between-group differences at each frequency. Each force level, being 15, 35, 55 and 70% maximal voluntary contraction (MVC), is presented as a separate subplot. The central dashed line is representative of equal coherence between testing groups. The between-group difference of coherence 95% confidence intervals (CI) are shown as horizontal, solid lines. Frequencies which cross the upper CI are representative of significantly greater IMC in the strength-trained group when compared to the dexterity-trained group. Similarly, frequencies which cross the lower CI represent greater coherence in the dexterity-trained group. Coherence bands are represented by gray shading, with the lightest shade identifying alpha ($\alpha$), and successively darker shades indicating beta ($\beta$) and gamma ($\gamma$) frequency bands. **B** Average values of pooled coherence across each coherence band, showing trends of coherence across force levels

Between-group differences in pooled coherence were observed at 15, 55 and 70% MVC (Fig. 6A). At 15% MVC, the strength-trained group showed greater IMC than dexterity-trained participants at a frequency within the alpha-band (13.02 Hz). At 55% MVC, a frequency in the gamma-band (56.42 Hz) was observed to be greater in strength-trained participants. At 70% MVC, the strength-trained group showed significantly greater coherence in frequencies within beta- (17.36 Hz) and gamma-bands (45.57 and 49.91 Hz), when compared to the dexterity-trained group.

### Post-hoc analysis of muscle activity

Visually identified “elbows” were observed in WCSS plots for the FDS muscle at 15% and 35% of MVC, indicating that clustering analysis was appropriate. Silhouette scores were subsequently calculated to identify the optimal number of clusters, with the highest value for both force levels occurring when $k = 2$ (15% MVC: SIL = 0.31; 35% MVC: SIL = 0.44). No “elbows" were visually observed in WCSS plots for the FDS at 55% and 70% MVC, nor in the APB at any force level, and thus, no further clustering analysis was performed.

**Fig. 7:**
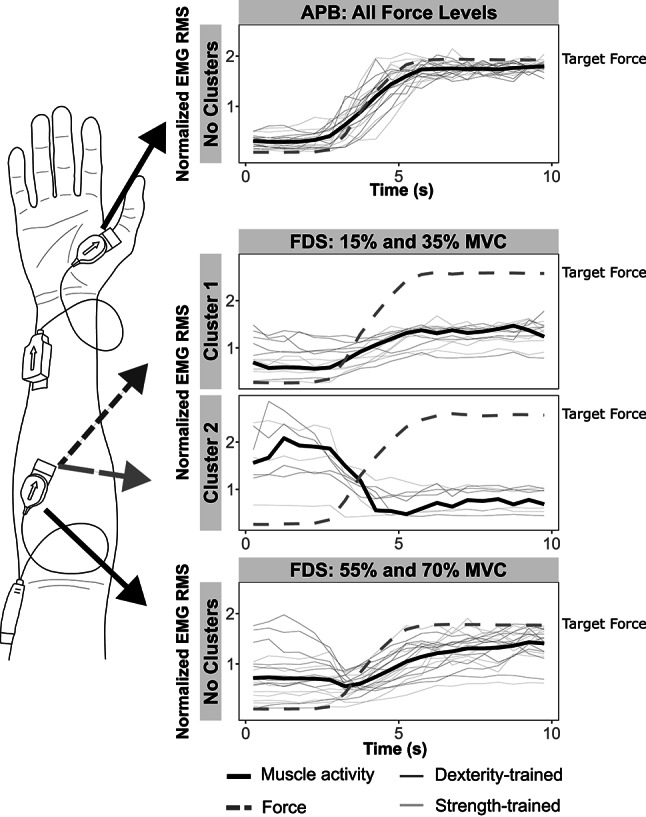
Cluster analysis of muscle activity during the submaximal five-finger pinch tasks. K-shape clustering analysis of the root-mean-square (RMS) of normalized electromyography (EMG) signals is presented for representative force trials. Representative data are shown for the abductor pollicis brevis (APB) muscle, which exhibited no identifiable clusters at any force level, and the flexor digitorum superficialis (FDS) muscle, which exhibited two clusters at 15% and 35% MVC, but no clusters at 55% and 70% MVC. Cluster-average muscle activity (cluster centroids) are displayed as black solid lines. Individual participant data are shown as fine solid lines, with testing groups differentiated by color. Force traces are overlaid as gray dashed lines, scaled to the target force

In the FDS at 15% MVC, “Cluster 1" contained 8 participants (6 strength- and 2 dexterity-trained) and “Cluster 2" contained 12 participants (4 strength- and 8 dexterity-trained). At 35% MVC, “Cluster 1" contained 13 participants (7 strength- and 6 dexterity-trained) and “Cluster 2" contained 7 participants (3 strength- and 4 dexterity-trained). Representative data of the clustering analysis are presented in Fig. 7.

## Discussion

The present study investigated the mechanical and neurophysiological differences subserving hand control in groups with different training histories. Given the large history of specialized hand training in each group, along with our observations of strength-trained participants producing larger maximal forces in the five-finger pinch task, and dexterity-trained participants being faster to complete a task requiring coordinated finger individuation, we suggest these groups were representative of individuals with distinct hand-control abilities. As noted, we observed differences in measures of five-finger pinch force, with the strength-trained participants producing both larger forces during an MVC, as well as steadier forces across submaximal force targets. Given the difference in force capacity remained after normalizing to forearm circumference and FDS thickness, the increased force capacity in strength-trained participants was not fully explained by increased muscle size. At submaximal forces, strength- and dexterity-trained groups differed in MU activity, with the most notable difference being higher average firing rates in the APB muscle of the dexterity-trained group at 35% MVC. Intermuscular coherence measures, estimating the shared synaptic inputs across APB and FDS muscles, were mostly inconsistent across force levels. However, large peaks of beta-band IMC were observed in dexterity- and strength-trained groups at opposite ends of the recorded force spectrum (15% and 70% MVC, respectively). Together, these results suggest differences in the force control and motoneuronal activity of strength- and dexterity-trained individuals.

Pinch force capacity was, as expected, significantly greater in the strength-trained group (Fig 3A). It is well accepted that force capacity is correlated with the hypertrophic response associated with chronic, progressive resistance training (Schoenfeld 2010; Hughes et al. 2018). The increased size of the structural components involved in the generation of force allows for increased force output through the relative increase in interaction between actin and myosin filaments (Huxley 1957; Taber et al. 2019). In the current study, however, normalizations of force to muscle size did not account for the total increase in force capacity observed in the strength-trained group. Both forearm circumference, being a gross measure of muscle cross-sectional area (with bone and non-contractile tissue), and FDS thickness, were used to normalize force between participants. Regardless of the normalization method, the strength-trained participants were able to produce greater force per unit muscle, with moderate to large effect. These findings indicate that the strength-trained group were able to produce larger forces than the dexterity-trained group, irrespective of muscle size. It is worth noting that resistance training may increase the functional capacity of muscle by improving its contractile components or fiber type, without increasing its size (Reggiani and Schiaffino 2020; Taber et al. 2019; Hughes et al. 2018). As the current study did not measure muscle composition, nor fiber type, we cannot dismiss the possibility of increased muscle force from contractile elements unrelated to neural drive. While this caveat should be considered, measures of muscle size in the current study did not describe the differences in force capacity. Given the moderate to large increases in force output in strength-trained participants, even when normalized to muscle size, changes in the neuromuscular system may explain a proportion of the variance between groups.

We hypothesized that the control of large forces would be steadier in the strength-trained participants. In a previous study, a group of elite rock-climbers were able to more steadily control finger-flexor force across all one second epochs of a fatiguing contraction at 80% MVC, when compared to untrained controls (Limonta et al. 2008). Additionally, several studies have shown increases in force steadiness following strength-training interventions in the upper- and lower-limb (Bilodeau et al. 2000; Hortobagyi et al. 2001; Keogh et al. 2019). However, these studies involved older cohorts or individuals with motor deficiencies. Given that our cohort consisted of young, motor-intact individuals, we also hypothesized that the dexterity-trained group would exhibit less force variation during low-force contractions. This was based on their regular exposure to skill-based practice of the hand, involving fine-force instrument manipulations. Indeed, skillful control of small forces has been observed in pianists and drummers (Oku and Furuya 2017; Fujii and Oda 2009), though in more training-specific and dynamic tasks than those observed here. In the present study, the strength-trained group were able to maintain forces with lower variation across all force levels, including relatively low force levels (15% MVC), with moderate effect (Fig. 3C). Given the higher force capacity of the strength-trained participants in our study, this could indicate low levels of force variability are associated with either training history or individual force capacity. Since the association between overall force capacity and force steadiness was weak to moderate across all force levels (Fig. 3D), accounting for 40% of variance at 15% MVC, and less than 1% of variance for all other intensities, our data suggest that the greater force steadiness displayed by strength-trained individuals was due to their training history, or habitual exposure to high-force tasks.

These behavioral findings prompted us to consider whether the differences in force steadiness across groups might reflect distinct neural control strategies. Specifically, we examined the firing behavior of MUs, as the central nervous system regulates force output through both the recruitment and firing rates of MUs (Enoka and Duchateau 2017). By comparing MU firing rates between groups, we aimed to explore whether training history influenced the neural drive to individual muscles during our experimental task. We observed higher MU firing rates in the APB for dexterity-trained individuals at 35% MVC (Fig. 4B). At 55% and 70% MVC, the dexterity-trained group also demonstrated slightly higher firing rates, although not reaching our required level of confidence. This may suggest a force-dependent increase in firing rate based on training history. The thumb of musicians is used for both stabilization or dexterous manipulations (Nolan and Eaton 1989), and therefore, may be required to maintain forces over a wider range of intensities during their practice. In contrast, MU firing rates in the FDS were statistically indistinguishable between groups at all measured force levels. Given the large number of degrees of freedom at the hand, and large the number of muscles which control them, recording firing rates from a greater number of muscles may identify changes in muscle coordination that could further explain the differences in force control between groups. Utilizing recordings from both surface and intramuscular HD-EMG arrays, which can allow decomposition algorithms to discern firings from a large number of MUs in both superficial and deep muscles (Grison et al. 2025), would allow for a deeper exploration of the changes in MU firing dynamics between these specialist-trained groups.

Given the observed differences between strength- and dexterity-trained groups in both force and MU control, changes in the common synaptic inputs to the motor neuron pools may also be expected. Although we identified frequencies with significant coherence across all submaximal intensities for both groups, we did not observe obvious differences that can be easily ascribed to training history-related changes in cortical or subcortical inputs to the motor neuron pools. In dexterity-trained participants, beta-band coherence, associated with corticomotor transmission (Baker et al. 1997; Conway et al. 1995; Power et al. 2006), was greatest at the lowest force level (15% MVC; Fig. 6B), with little significant coherence observed at higher force levels (Fig. 5A). The trend was reversed in strength-trained participants, for whom the greatest beta-band coherence was observed at the highest force level (70% MVC). Beta-band frequencies have been suggested to reflect synchronized communication between cortex, muscle, and back to cortex (Baker 2007; Engel and Fries 2010; Aumann and Prut 2015), possibly involved in the synergistic control of multiple muscles (Reyes et al. 2017; Aumann and Prut 2015). To the extent that these mechanisms underpin the beta-band coherence we observed, our results suggest that participants relied more heavily upon cortical output when force demands were most consistent with their training history. We also observed large peaks of gamma-band coherence, previously associated with corticomotor transmission at high force levels (Brown 2000; Omlor et al. 2007; Mima et al. 1999), in both groups at 70% MVC. Between-group differences within this band were also observed at 55% and 70% MVC, with strength-trained participants showing significantly greater coherence at frequencies within the range of $45-60$ Hz (Fig. 6A). These frequencies align with the Piper Rhythm - a phenomenon in which MU spike trains commonly occur in the $40-60$ Hz range during steady contractions, and which is thought to be driven by activity in the contralateral motor cortex (Brown et al. 1998; Brown 2000). In the alpha-band, frequencies associated with subcortical inputs (Grosse and Brown 2003), our findings of IMC showed no consistent trends within, nor between groups.

While beta- and gamma-band coherence showed some group- and force-dependent differences, these high-frequency oscillations are unlikely to account for the observed differences in force steadiness across groups because such frequencies exceed the functional bandwidth for force modulation (Farina et al. 2014; Negro et al. 2009). However, the greater force variability demonstrated by dexterity-trained participants likely reflects more variable low-frequency common inputs to the target muscles’ motor neuron pools, as the coefficient of variation of force is thought to approximate variability in this “common drive” (Enoka and Farina 2021). Due to the low number of MUs identified in the FDS muscle (Fig. 4A), analysis of the coherence between cumulative spike trains of both muscles, which can identify the slow oscillatory components related to force control (Enoka and Farina 2021; Farina and Negro 2015), was not possible. Although significant coherence was detected $<8$ Hz in the compound EMG signals, these do not accurately reflect the low-frequency common inputs across motor neuron pools (Dideriksen et al. 2018). Future research should consider identifying coherence in these low frequency ranges, which may provide further insight into neuromuscular changes with training history.

During data analysis, visual inspection revealed variations in FDS muscle activity patterns across participants during the initial force ramp period. To assess whether participants could be grouped based on these variations, a post-hoc clustering analysis was undertaken. Two distinct patterns of muscle activity were identified in the FDS during low-intensity contractions (15% and 35% MVC): a pattern whereby muscle activity followed the shape of the force trace (Cluster 1), and a pattern inversely related to the force trace (Cluster 2; Fig. 7). Given the large number of redundant degrees of freedom involved in hand control (Santello et al. 2013), these findings may suggest the muscle coordination for producing the required finger-flexion varied across participants. Although the strategies were not constrained to a specific group, a greater number of strength-trained participants utilized the force-related strategy (Cluster 1) than dexterity-trained. At higher force levels (55% and 70% MVC), clusters of participants with different FDS muscle activity were no longer identified. Therefore, as force requirements increased, participants with previously different strategies began to utilize the FDS muscle to produce force in a similar way. Both MU and IMC analyses were calculated from only the steady portion of each trapezoidal contraction, where the influence of changes in muscle activity during the ramp period were excluded. However, their findings should be considered in the context of between-participant differences in FDS activity patterns at low force levels.

The current study had several limitations which must be considered when interpreting its findings. The study recruited a small number of participants, including only a single female participant in each group. Although there is evidence for sex-based differences in the neuromuscular system, removal of female-participant data did not affect the interpretation of results. The present study only measured the muscle thickness of a single extrinsic muscle (the FDS). Although forearm circumference and FDS thickness are correlated with force capacity (Abe et al. 2015; Anakwe et al. 2007), future studies should consider the measurement of all muscles of the forearm and hand to provide a greater comparison of muscular structure and its effect on hand force. Additionally, given each muscle involved in hand control may have task-specific roles, exploring these measures in a larger number of muscles would provide a broader understanding of the shared synaptic inputs and MU control. For example, implementing the framework set out by Tanzarella et al. (2020) would provide robust measures from a large sample of hand muscles. The current study utilized a simple, isometric five-finger pinch-grip task to identify differences in force capacity and neuromuscular control. This task was chosen to ensure contraction from all digits. Whilst we suggest the movement is similar to the functional grasping movements of daily living, and those required in the training of both groups, it may have biased the synchronous, isometric digit control strategies which are commonly used by strength-trained individuals. While isometric tasks are optimal for MU decomposition (Yokoyama et al. 2021), they do not allow insight into the control of dynamic movements which constitute the majority of daily hand function and musical training. Future studies should consider using high-density surface EMG, which may allow the decomposition of MU spike trains during more complex isotonic movements (Oliveira and Negro 2021). It is also important to note that, when considering the entire coherence spectrum, our effects were small and resulted from a small number of trials containing significant coherence in each band (Fig. 5B). Intermuscular coherence, especially when estimated from EMG signals, can be affected by task-related variables (Riddle and Baker 2006), crosstalk between electrodes, and signal preprocessing procedures. We attempted to increase the reliability of this measure with task-related constraints, such as reducing the impact of visuomotor and force-fluctuation variability by choosing only the steadiest portion of data for analysis, standardizing the arm and hand position, and ensuring large distances between recording electrodes. Despite these standardizations, we observed between-participant differences in FDS muscle activity at both 15% and 35% MVC, which may reflect different force generation strategies. The hand and wrist was unable to be constrained in the current study, and thus, small changes in wrist and finger position between trials and participants is likely. Additional IMC analysis on the cluster of participants using only the expected FDS muscle activity pattern (positive EMG-force relationship; Cluster 1: Fig. 7) predominantly showed amplified peaks of coherence in the same bands as those observed from analysis of the entire dataset, supporting our main conclusions (results from this additional analysis are presented in Online Resource 2). However, considering a detailed examination of the kinematics and kinetics of the hand, or constraining the hand position may provide the basis for more consistent MU behavior. Finally, we would like to note that preprocessing methods, particularly when comparing raw and rectified EMG signals, substantially affected our resultant coherence estimates. There has been some debate in the literature regarding the preprocessing of EMG signals prior to coherence analysis (Ward et al. 2013; Farina et al. 2013; McClelland et al. 2012; Boonstra and Breakspear 2012), and future research should carefully consider their implementation of preprocessing procedures when calculating IMC from global EMG signals.

## Conclusion

In conclusion, our results suggest that the training history of hand muscles may affect force control, along with the inputs to and outputs of MUs. Specifically, steady control of hand force may be reliant on a training history involving high forces, rather than gross force capacity. We did not identify obvious changes to the neural substrates of motoneuronal inputs to hand muscles between individuals with dexterous or high- force training histories. However, small changes in high-frequency, beta- and gamma-band common inputs, and motor neuron outputs may indicate differences in the contribution of descending neural drive from cortical motor regions.

## Supplementary Information

Below is the link to the electronic supplementary material.

Supplementary file 1.

Supplementary file 2.

## Data Availability

Data is available by request, please contact the corresponding author. All data analysis code is available at the GitHub repository: https://github.com/dyljcarter/repo-carter-et-al-2025
