# Supplementary material for: Neuromuscular control of a five-finger pinch task is influenced by training history
Source: Exp Brain Res. 2025 Sep 2;243(10):205. doi: 10.1007/s00221-025-07147-z (PMC12405043; doi:10.1007/s00221-025-07147-z)
Supplement: Supplementary file 1 [file 221_2025_7147_MOESM1_ESM.pdf]

Online Resource 1

Article Title: Neuromuscular control of a five-finger pinch task is influenced by training history.
Journal: Experimental Brain Research

Authors: Dylan J. Carter[1,2*], James R. Forsyth[2], Joshua P. M. Mattock[2], Jonathan Shemmell[1]

[1]Neuromotor Adaptation Laboratory, University of Wollongong. School of Medical, Indigenous, and Health Sciences. Faculty of Science, Medicine, and Health, Northfields Avenue, Wollongong, 2522, NSW, Australia.
[2]Biomechanics Research Laboratory, University of Wollongong. School of Medical, Indigenous, and Health Sciences. Faculty of Science, Medicine, and Health, Northfields Avenue, Wollongong, 2522, NSW, Australia.

[*]**Corresponding Author**: Dylan J. Carter dcarter@uow.edu.au

Strength-trained

MVC Level	Alpha-band Frequencies (Hz)	Beta-band Frequencies (Hz)	Gamma-band Frequencies (Hz)
**15%**	13.02	–	41.23
**35%**	–	–	36.89<br>45.57
**55%**	10.85<br>15.19	23.87	56.42
**70%**	–	17.36<br>19.53	45.57<br>49.91

Table 1: Significant frequencies identified in the pooled coherence analysis for the strength-trained group.

Dexterity-trained

MVC Level	Alpha-band Frequencies (Hz)	Beta-band Frequencies (Hz)	Gamma-band Frequencies (Hz)
**15%**	–	19.53	32.55
		21.70	34.72
			36.89
**35%**	10.85	–	32.55
	13.02		47.74
**55%**	13.02	17.36	32.55
			36.89
			43.40
			49.91
**70%**	10.85	–	36.89
			43.40

Table 2: Significant frequencies identified in the pooled coherence analysis for the dexterity-trained group.
