# Supplementary material for: Neuromuscular control of a five-finger pinch task is influenced by training history
Source: Exp Brain Res. 2025 Sep 2;243(10):205. doi: 10.1007/s00221-025-07147-z (PMC12405043; doi:10.1007/s00221-025-07147-z)
Supplement: Supplementary file 2 [file 221_2025_7147_MOESM2_ESM.pdf]

Online Resource 2

Article Title: Neuromuscular control of a five-finger pinch task is influenced by training history.
Journal: Experimental Brain Research

Authors: Dylan J. Carter[1,2*], James R. Forsyth[2], Joshua P. M. Mattock[2], Jonathan Shemmell[1]

[1]Neuromotor Adaptation Laboratory, University of Wollongong. School of Medical, Indigenous, and Health Sciences. Faculty of Science, Medicine, and Health, Northfields Avenue, Wollongong, 2522, NSW, Australia.
[2]Biomechanics Research Laboratory, University of Wollongong. School of Medical, Indigenous, and Health Sciences. Faculty of Science, Medicine, and Health, Northfields Avenue, Wollongong, 2522, NSW, Australia.

[*]**Corresponding Author**: Dylan J. Carter dcarter@uow.edu.au

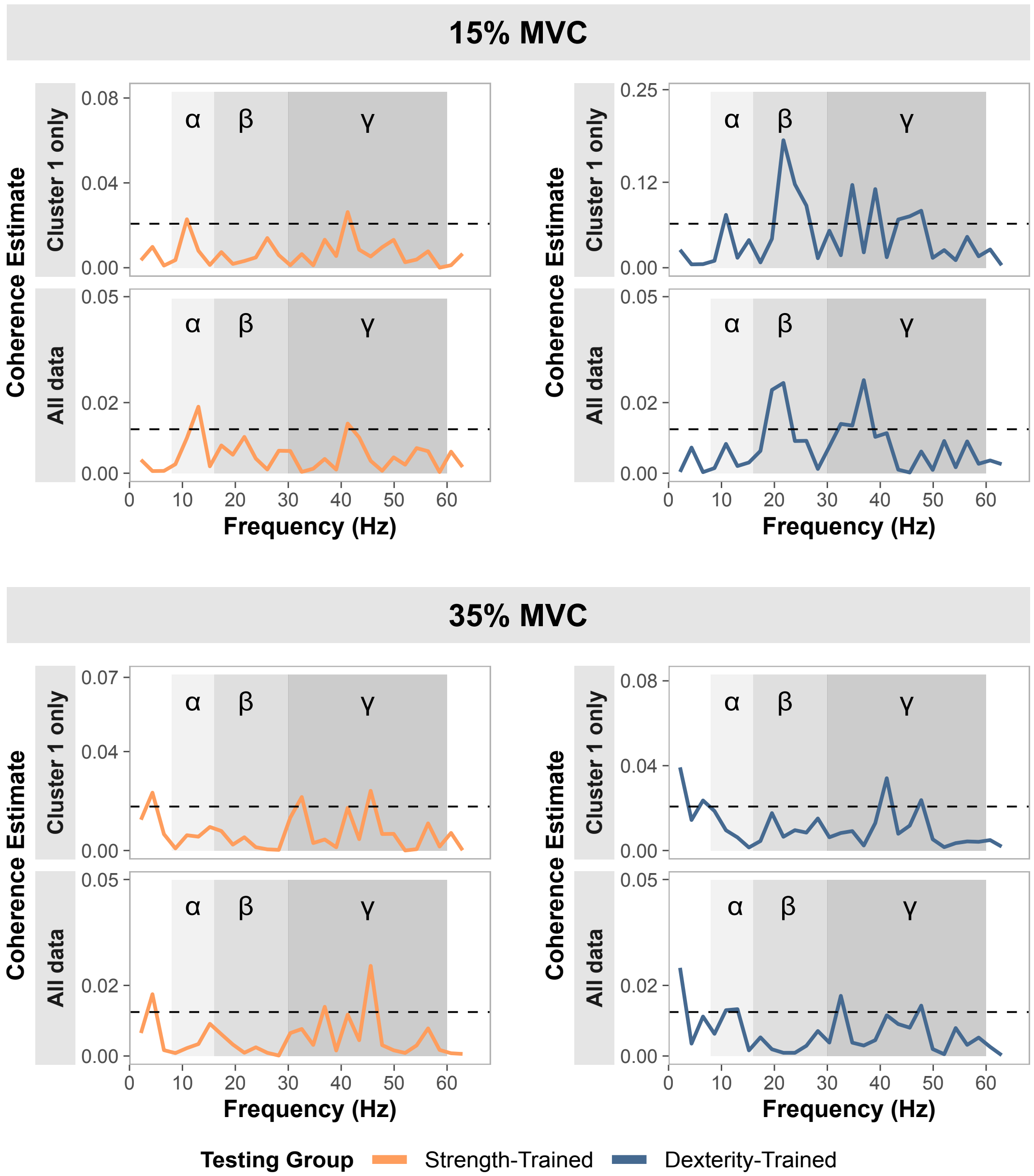

Figure 1: Comparison of coherence estimates at 15% and 35% maximal voluntary contraction (MVC). Presented are coherence estimates, calculated using identical methods to those described in our Methods section, from the subset of participants using the "Cluster 1" muscle strategy (positive force-EMG relationship), and compared against estimates calculated from the entire dataset. For "Cluster 1", at 15% MVC: dexterity-trained $N = 2$, strength-trained $N = 6$; at 35% MVC: dexterity-trained $N = 6$, strength-trained $N = 7$. The 95% confidence limit (CL) is represented by a dashed, horizontal line. The number of participants using the "Cluster 1" strategy differed for each force level and testing group, which results in differences in the magnitude of coherence estimates. To facilitate comparisons of data relative to the CL, the y-axes of each subplot have been individually scaled so that CLs are aligned at the same visual height across subplots. The coherence bands are represented by gray shading, with alpha- (α), beta- (β) and gamma-bands (γ).
